# A genetic variant of long non-coding RNA TINCR is associated with psoriasis in the Turkish population^☆^

**DOI:** 10.1016/j.clinsp.2026.101189

**Published:** 2026-09-30

**Authors:** Egemen Akgun, Fadime Mutlu Icduygu, Burak Aksan, Isil Deniz Oguz, Sevgi Kulakli, Ebru Alp

**Affiliations:** a Department of Medical Biology, Faculty of Medicine, Giresun University, Giresun, Turkey; b Department of Medical Genetics, Faculty of Medicine, Giresun University, Giresun, Turkey; c Department of Dermatology, Faculty of Medicine, Giresun University, Giresun, Turkey

**Keywords:** TINCR, H19, lncRNA, Psoriasis, Polymorphism

## Abstract

**Background:**

TINCR and H19 are long non-coding RNAs known to be involved in keratinocyte proliferation and differentiation. The objective of this study was to investigate the contribution of *TINCR* rs2288947 and *H19* rs217727 polymorphisms to the pathogenesis of psoriasis.

**Methods:**

The study group comprised 284 patients with psoriasis and 253 healthy controls. Real-time PCR method was used for genotyping of rs2288947 and rs217727 polymorphisms.

**Results:**

In rs2288947 polymorphism, AA genotype (BH-adjusted *p* = 0.014, adjusted OR = 1.92, 95% CI 1.20‒3.09), combined genotypes GA+AA (BH-adjusted *p* = 0.021, adjusted OR = 1.64, 95% CI 1.10‒2.45) and A allele (BH-adjusted *p* = 0.014, adjusted OR = 1.39, 95% CI 1.10‒1.76) showed a significant association with psoriasis. For H19 rs217727, results were inconclusive, reflecting limited precision of the effect estimates in our study cohort (overall genotype distribution, *p* = 0.425), and despite high genotyping quality, control genotype distributions deviated from Hardy–Weinberg equilibrium.

**Conclusion:**

Our findings indicate that the *TINCR* rs2288947 polymorphism may be associated with an elevated risk of psoriasis in this Turkish cohort. H19 rs217727 showed no significant association; given the breadth of the confidence intervals, this result is best viewed as inconclusive. Independent replication and functional assays will help clarify biological relevance.

## Introduction

Psoriasis is a chronic inflammatory dermatological condition that is triggered by a combination of genetic, environmental, and immunological factors. Affecting >125 million people worldwide, psoriasis is equally prevalent in men and women.^1^ There are five clinical types of psoriasis, the most common of which is chronic plaque psoriasis. Other types include guttate, erythrodermic, inverse, and pustular psoriasis.^2^ The pathogenesis of psoriasis is complex and is the result of inflammation triggered by interactions between immune cells, keratinocytes, and other skin cells.^3^, ^4^, ^5^ Derived from stem cells in the stratum basale of the epidermis, keratinocytes undergo differentiation during their migration through the layers of the epidermis, accompanied by a gradual decline in their proliferative capacity. Eventually, they die to become part of the stratum corneum, the outermost layer of the epidermis.^4^^,^^6^ Patients with psoriasis typically exhibit thickening of the epidermis, a condition known as acanthosis, which is largely attributed to accelerated proliferation and poor differentiation of keratinocytes.^4^^,^^7^ There is also a strong genetic basis for the disease. Meta-analyses of Genome-Wide Association Studies (GWAS) for psoriasis have reported >100 psoriasis susceptibility loci.^8^ However, a notable proportion of the signals obtained in GWAS studies are in non-coding DNA fragments, indicating that epigenetic regulators, such as microRNAs (miRNAs) and long non-coding RNAs (lncRNAs), may play a role in the disease.^9^ Indeed, many studies have shown a significant association between non-coding RNAs and psoriasis.^10^, ^11^, ^12^, ^13^, ^14^ lncRNAs are defined as non-protein-coding RNA transcripts exceeding 200 nucleotides in length. lncRNAs represent the most prevalent type of non-coding RNA, comprising over 100,000 transcripts in the human genome. They play a multitude of diverse roles, as evidenced by their involvement in a range of biological processes.^15^^,^^16^ lncRNAs can regulate gene expression at any stage (epigenetic, during transcription, or post-transcriptional) through interactions with RNA, DNA, and proteins.^17^ These properties afford lncRNAs a pivotal role in the realization of essential biological functions, including cell growth, differentiation, migration, apoptosis, and the cell cycle.^15^, ^16^, ^17^, ^18^

Tissue differentiation-induced Non-Coding RNA (TINCR), which produces a transcript approximately 3.7 kb in length, is located on chromosome 19 and is highly expressed in stratified epithelial tissues and especially in differentiated human epithelial keratinocytes.^18^^,^^19^ TINCR regulates the expression of genes involved in epidermal differentiation through post-transcriptional mechanisms.^18^ The TINCR transcript binds to the mRNAs of genes involved in epithelial differentiation using its 25-bp long TINCR Box motif, thereby regulating differentiation. Additionally, it stabilizes mRNAs involved in differentiation by directly binding to Staufen1 (STAU1) protein.^18^ Collectively, these findings suggest that TINCR may be a crucial component in somatic differentiation after development.

The human H19 long non-coding RNA is localized on chromosome 11p15.5 and plays a critical role in the pathogenesis of chronic inflammatory diseases, including cardiovascular disease,^20^ atherosclerosis,^21^ osteoarthritis,^22^ and collagen-induced arthritis.^23^ Additionally, two recent studies have demonstrated an association between psoriasis and H19 lncRNA.^24^^,^^25^

Since a single nucleotide change in the sequence of a target region can affect lncRNA regulation, we aimed to investigate the contribution of the rs2288947 and rs217727 polymorphisms of TINCR and H19 lncRNAs, respectively, which are involved in keratinocyte proliferation and differentiation, to the development of psoriasis.

## Materials and methods

### Study population

The study included 284 individuals with a confirmed diagnosis of psoriasis as the patient group and 253 healthy individuals without a history of psoriasis as the control group. Psoriasis was diagnosed by the physicians of the Giresun University Faculty of Medicine Training and Research Hospital Dermatology Clinic, and it was ensured that the individuals included in the study as the patient group did not have any autoimmune or systemic inflammatory disease. The control group was selected from individuals who did not have any autoimmune or systemic inflammatory disease and who did not have a family history of psoriasis and who applied to the Giresun University Faculty of Medicine Training and Research Hospital for regular health check-ups. All participants were of self-reported Turkish ethnic origin.

### Ethical considerations

The study protocol was conducted in accordance with the Declaration of Helsinki and approved by Giresun University Clinical Research Ethics Committee (approval date: March 18, 2021; study protocol/decision number: 08). Participants (or their parents/legal guardians/relatives) were asked to sign a written informed consent form.

This observational case-control study was designed and reported in accordance with the Strengthening the Reporting of Observational Studies in Epidemiology (STROBE) guidelines.

### Genotyping of rs2288947 and rs217727 polymorphisms

DNA isolation was performed from venous blood using a high-purity PCR template preparation kit, which was commercially available, and in line with the manufacturer's instructions (Roche Diagnostics, Mannheim, Germany). Following isolation, DNA concentration and purity were measured spectrophotometrically, and DNA samples were stored at −20 °C for future use. Genotyping of rs2288947 and rs217727 polymorphisms was performed by real-time PCR using the LightCycler® FastStart DNA Master HybProbe kit (Cat. no. 12239272001, Roche, Mannheim, Germany). The sequences of the primers and probes utilized are as follows: F; 5′-CCTAGAGGCCAGAAGCATAAG, R; 5′-GGGTACTGGCTGAAGGAATAG, FL; 5′-CTACAATTACTTACCTGGAGTGAGATCTAGG-FL, LC; 5′-red640-TCCCgGGAGGCAGGAGAA-Ph for rs2288947 and F;5′-AAGAAGAAGAAGAGGGAGGTGGT, R; 5′-CTAGGAACCAGACCAGACCTCATCA, FL; 5′-AGCTCTGGGATGATGTGGTGGCTGGTGGT-FL, LC; 5′-red640-GTCAACCGTCCgCCGCA-Ph for rs217727. Real-time PCR cycling was performed on a LightCycler 480 II instrument (Roche, Mannheim, Germany) as follows: 95 °C for 10 min, followed by 60 cycles of 95 °C for 5 s, 60 °C for 10 s, 50 °C for 10 s (ramp rate 20 °C/s) and 72 °C for 11 s, followed by 95 °C for 20 s, 32 °C for 20 s and melt curve analysis at 80 °C for 0 s (ramp rate 0.2 °C/s). Genotyping was performed using standardized procedures without knowledge of case-control status.

### Genotyping quality control

We pre-specified melt-curve quality thresholds (Resolution ≥ 0.60; Score ≥ 0.90), summarized call rates at plate and study level, and tested case-control differential missingness (Fisher’s exact). Control-group Hardy-Weinberg Equilibrium (HWE) was assessed using exact and mid-P tests; for transparency, we provide genotype counts, expected counts, observed/expected heterozygosity (Hobs/Hexp), and the inbreeding coefficient (*F*) in Supplementary Table S5. Detailed genotyping quality-control outputs (per-plate call counts, case-control differential missingness, flagged low-quality calls, Hardy-Weinberg metrics, and representative melting-peak plots) are provided in Supplementary Figures S1 and S2 and Supplementary Tables S1–S5.

### Statistical analysis

The statistical analyses were conducted using the IBM SPSS Statistics® software, version 25 (IBM Corp., Armonk, NY, USA). Continuous variables are presented as mean ± standard deviation, while categorical variables are expressed as number (percentage). Hardy-Weinberg equilibrium was assessed in controls using both exact and mid-P tests. For consistency, HWE *p*-values reported in the main text refer to the exact test. The Mann-Whitney *U*-test or Student's *t*-test was employed to assess the significance of differences in continuous variables between groups. The Chi-Square test was employed to compare genotype and allele frequencies between patient and control groups, with Odds Ratio (OR) and Confidence Interval (95% CI) calculations conducted according to the reference genotype. To analyze the association between genotypes and clinical features, chi-square tests were utilized for categorical variables and one-way ANOVA tests were employed for continuous variables. Logistic regression analysis was conducted to analyze the independent effect of polymorphisms from other risk factors. All models were adjusted for potential confounding variables, including age, sex, Body Mass Index (BMI), smoking status (yes/no), and presence of comorbidities (e.g., hypertension, diabetes, cardiovascular diseases), which were coded as binary or continuous variables as appropriate. We report adjusted Odds Ratios (ORs) with 95% confidence intervals and two-sided p-values. Within cases, disease severity was evaluated using both a continuous outcome and a binary outcome. The continuous analysis used ln[PASI+1] as the dependent variable with Huber-White robust standard errors (SPSS GENLIN; Normal, Identity). In addition, a binary severity outcome (PASI ≥ 5 vs. < 5) was analyzed using logistic regression. The same covariate set was used in both models, including age, sex, body mass index, smoking status, and comorbidities. The PASI ≥ 5 threshold was pre-specified to distinguish mild from at least moderate disease severity and was not selected in a data-driven manner. To address multiple testing, the Benjamini-Hochberg False Discovery Rate (BH-FDR) correction was applied. The correction was applied separately within each SNP across the model-specific association tests (GA vs. GG, AA vs. GG, GA+AA vs. GG, and allelic comparison). The overall chi-square test of genotype distribution was reported separately and was not included in the BH correction set. Statistical significance was set at α = 0.05. No formal a priori sample size or power calculation was performed due to the exploratory design of the study, and the sample size was determined by the number of available participants.

### Bioinformatic analysis

To explore the potential regulatory effects of rs2288947 on TINCR expression, publicly available expression Quantitative Trait Loci (eQTL) data from the GTEx Portal (https://gtexportal.org) were utilized. Genotype-dependent TINCR expression levels in non-sun-exposed (suprapubic) skin tissue were visualized using violin plots. Normalized expression values were compared across genotypes, and associated *p*-values were reported as provided by the GTEx database (Fig. 1).

**Fig. 1:**
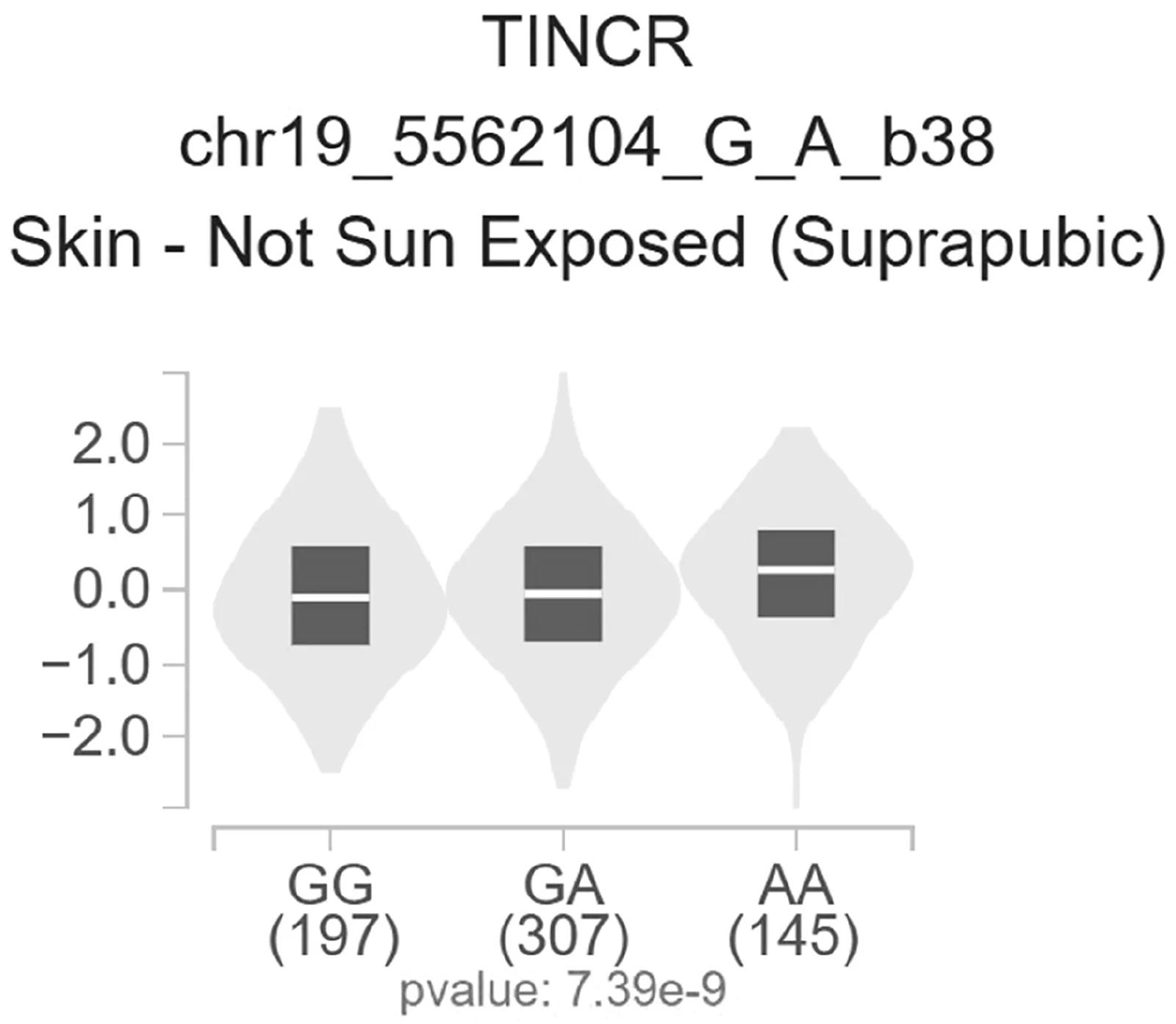
GTEx-derived genotype-dependent expression levels of TINCR in non-sun-exposed (suprapubic) skin tissue according to rs2288947 genotype. The y-axis represents GTEx-normalized expression values derived from RNA-seq expression quantification based on Transcripts Per Million (TPM), as provided by the GTEx Portal; these values should be interpreted as relative normalized expression rather than raw TPM. Individuals carrying the A allele (GA and AA genotypes) show increased TINCR expression compared with GG homozygotes. Expression differences were statistically significant (*p* = 7.39×10^–9^). Data derived from the GTEx Portal (v8).

To assess whether rs2288947 affects alternative splicing of TINCR, publicly available splicing Quantitative Trait Loci (sQTL) data from the GTEx Portal (https://gtexportal.org) were also examined. Splicing patterns in skin tissue (Not Sun Exposed – Suprapubic) were analyzed based on the relative abundance of transcript isoforms associated with different genotypes. Violin plots were used to visualize genotype-specific differences in intron excision ratios or exon inclusion levels. The statistical significance of splicing variation across genotypes was reported using the *p*-values provided by the GTEx database (Fig. 2).

**Fig. 2:**
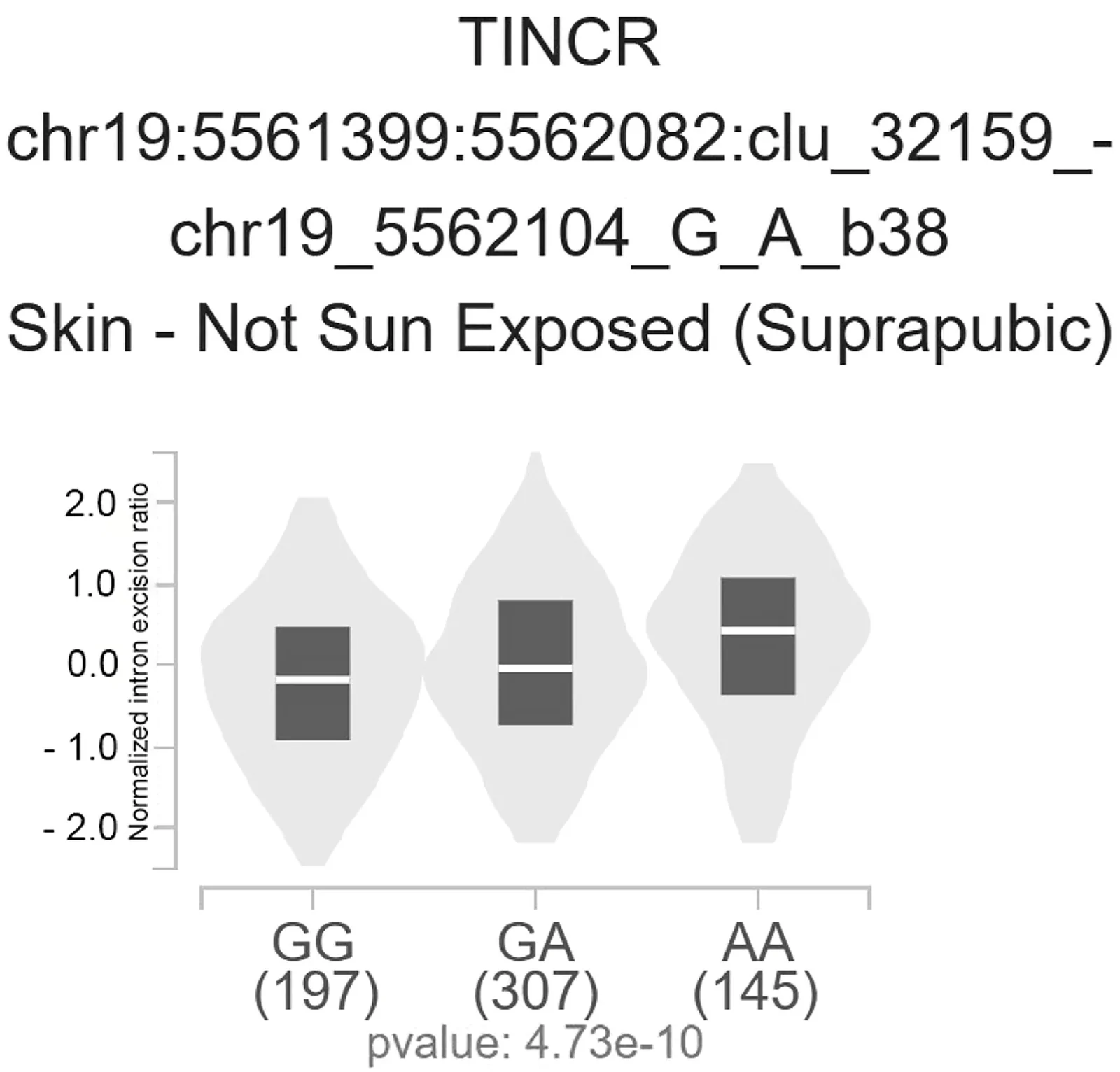
GTEx-derived splicing Quantitative Trait Locus (sQTL) signal for rs2288947 in non-sun-exposed (suprapubic) skin. The y-axis represents the intron excision ratio, defined as the normalized proportion of splice-junction reads supporting use/excision of the indicated intron relative to the total junction reads in the corresponding intron cluster, as provided by the GTEx/LeafCutter framework. rs2288947 was associated with an altered intron excision ratio for TINCR (*p* = 4.73×10^–10^). Data derived from the GTEx Portal (v8).

## Results

Our study population consisted of a total of 537 individuals, of which 284 were patients and 253 were controls. Demographic and clinical characteristics of the study population are shown in Table 1. The mean ages in the psoriasis and control groups were 45.22 ± 14.45 and 37.11 ± 16.36 years, respectively (*p* < 0.001). A positive family history was observed in 123 (43.3%) of the individuals with psoriasis. The mean age at onset was 30.32 ± 15.63 years, the mean disease duration was 15.74 ± 12.43 years, and the mean PASI score was 8.21 ± 8.48.

**Table 1:** Demographic and clinical characteristics of the patient and control groups.

**Parameters**	**Cases (%),** ***n* = 284**	**Controls (%),** ***n* = 253**	*p***-value**
Age (years), (mean ± SD)	45.22 ± 14.45	37.11 ± 16.36	<0.001^a^
Gender, n (%)			
Female	149 (52.5)	156 (61.7)	0.032^a^
Male	135 (47.5)	97 (38.3)	
Age of onset (years)	30.32 ± 15.63		
Disease duration (years), (mean ± SD)	15.74 ± 12.43		
Positive family history, n (%)	123 (43.3)		
Psoriatic arthritis, n (%)	69 (24)		
PASI	8.21 ± 8.48		

a *p* < 0.05.

Genotype and allele frequencies of TINCR rs2288947 and H19 rs217727 polymorphisms in psoriasis and control groups are shown in Table 2. Genotypic distributions of the TINCR rs2288947 polymorphism showed statistically significant differences between psoriasis patients and healthy controls (χ^2^*p* = 0.030). Logistic regression analysis revealed that individuals carrying the AA genotype had a significantly increased risk of psoriasis compared to those with the GG genotype (adjusted OR = 1.92, 95% CI: 1.20–3.09, *p* = 0.007; BH-adjusted *p* = 0.014). Similarly, a significant association was observed under the dominant genetic model (GA+AA vs. GG) (adjusted OR = 1.64, 95% CI: 1.10–2.45, *p* = 0.016; BH-adjusted *p* = 0.021). The allelic comparison also revealed a significant association, with the A allele being associated with higher odds of psoriasis relative to the G-allele (adjusted OR = 1.39, 95% CI: 1.10–1.76, *p* = 0.006; BH-adjusted *p* = 0.014). However, the GA genotype alone did not reach statistical significance compared to GG (adjusted OR = 1.50, 95% CI: 0.93–2.40, *p* = 0.094; BH-adjusted *p* = 0.094). Overall, the effect sizes for TINCR were modest and are interpreted with caution. The analysis of the relationship between genotype distributions and clinical characteristics in the patient group revealed no significant correlation with disease duration, age at onset of the disease, or positive family history (*p* > 0.05). In the adjusted binary logistic regression model, rs2288947 showed a borderline association with the binary PASI outcome (PASI ≥ 5 vs. < 5) under the dominant model (GA+AA vs. GG; adjusted OR = 0.570, 95% CI: 0.326–0.998, *p* = 0.049). However, this finding was not confirmed when PASI was modeled as a continuous outcome (ln[PASI+1]) using the same covariate set with robust standard errors (β = 0.035, 95% CI: −0.093 to 0.163, per-allele *p* ≈ 0.589). Therefore, the severity findings were not robust across models and were interpreted as exploratory.

**Table 2:** Genotype and allele distribution of SNPs in psoriasis patients and control group individuals.

**Genotype**	**Cases (%) *n* = 284**	**Controls (%) *n* = 253**	**χ^2^***p***-value**	**OR (95% CI)**	*p***-value**	**Adjusted OR (95% CI)**	**Adjusted***p***-values**	**BH Adjusted***p***-value**
rs2288947			0.030^a^					
GG	85 (29.9)	101 (39.9)		1^a^				
GA	96 (33.8)	82 (32.4)		1.39 (0.92–2.10)	0.116	1.50 (0.93–2.40)	0.094	0.094
AA	103 (36.3)	70 (27.7)		1.75 (1.15–2.66)	0.009^a^	1.92 (1.20–3.09)	0.007^a^	0.014^a^
GA+AA	199 (70.1)	152 (60.1)		1.55 (1.09–2.22)	0.015^a^	1.64 (1.10–2.45)	0.016^a^	0.021^a^
Alleles			0.002^a^					
G	266 (46.8)	284 (56.1)		1^a^				
A	302 (53.2)	222 (43.9)		1.45 (1.14–1.85)	0.002^a^	1.39 (1.10–1.76)	0.006^a^	0.014^a^
**Genotype**	**Cases (%) n = 284**	**Controls (%) n = 253**	**χ^2^***p***-value**	**OR (95% CI)**	*p***-value**	**Adjusted OR (95% CI)**	**Adjusted***p***-values**	**BH Adjusted***p***-value**
rs217727			0.425					
GG	127 (44.7)	107 (42.3)		1^a^				
GA	110 (38.7)	93 (36.8)		0.99 (0.69–1.45)	0.986	0.95 (0.62–1.45)	0.801	0.801
AA	47 (16.6)	53 (20.9)		0.75 (0.47–1.20)	0.223	0.82 (0.48–1.40)	0.457	0.631
GA+AA	157 (55.3)	146 (57.7)		0.91 (0.64–1.28)	0.571	0.81 (0.49–1.33)	0.404	0.631
Alleles			0.249					
G	364 (64.1)	307 (60.7)		1^a^				
A	204 (35.9)	199 (39.3)		0.86 (0.68–1.11)	0.249	0.91 (0.70–1.18)	0.473	0.631

a *p* < 0.05. OR, Odds Ratio; CI, Confidence Interval; BH, Benjamini-Hochberg correction; 1^a^, Reference genotype/allele. Adjusted odds ratios and *p*-values were derived from multivariate logistic regression models controlling for age, sex, BMI, smoking status, and comorbidities. BH-adjusted *p*-values were calculated separately for each SNP across the model-specific association tests (GA vs. GG, AA vs. GG, GA+AA vs. GG, and allelic comparison); the overall Chi-Square test of genotype distribution was reported separately and was not included in the BH correction set.

In contrast, the H19 rs217727 polymorphism did not show a statistically significant association with psoriasis in any of the tested models. The chi-square test for genotypic distribution yielded a *p*-value of 0.425. Logistic regression analysis revealed no significant association in the genotypic (AA vs. GG: adjusted OR = 0.82, *p* = 0.457; BH-adjusted *p* = 0.631), dominant (GA+AA vs. GG: adjusted OR = 0.81, *p* = 0.404; BH-adjusted *p* = 0.631), or allelic (A vs. G: adjusted OR = 0.91, *p* = 0.473; BH-adjusted *p* = 0.631) models.

The observed frequencies of rs2288947 and rs217727 in the control groups did not align with the HWE expectations (two-sided exact test, *p* < 0.001). The control-group deviations at both loci reflected heterozygote deficits (rs2288947: Hobs = 0.324 vs. Hexp = 0.492; rs217727: Hobs = 0.368 vs. Hexp = 0.477; Supplementary Table S5). Accordingly, we report pre-specified, QC-oriented, model-preserving diagnostics to evaluate the possibility of technical artifacts. Study-wide call rates were high (rs2288947: 537/554, 96.9%; rs217727: 537/555, 96.8%) (Supplementary Tables S1 and S2), case–control differential missingness was not detected (*p* = 0.309 and *p* = 0.633, respectively) (Supplementary Table S3), and representative melting-peak plots showed three cleanly separated genotype clusters (Supplementary Figs. S1 and S2). Although these genotyping Quality-Control (QC) indicators reduce the likelihood of a pervasive genotyping artifact, residual genotype misclassification or other systematic assay-related error cannot be completely excluded. Because departures from HWE can also arise from sampling variation or subtle population structure (among other genetic factors),^26^ we interpret the associations with appropriate caution. Additional QC details, including lists of flagged low-quality calls, are provided in Supplementary Tables S4a and S4b.

## Discussion

The objective of our study was to investigate whether TINCR rs2288947 and H19 rs217727 polymorphisms contribute to the development of psoriasis in the Turkish population. To the best of our knowledge, this is the first study to investigate the association between psoriasis and TINCR rs2288947 and H19 rs217727 polymorphisms.

The available evidence increasingly suggests that lncRNAs play a pivotal role in cell proliferation and differentiation.^27^ While the effects of lncRNAs on skin biology remain largely unknown, previous studies have shed light on the significant roles of TINCR and H19 lncRNAs in this context.^18^^,^^24^^,^^25^^,^^28^ Kretz et al. reported that TINCR lncRNA production in human keratinocyte cells increases >150-fold during epidermal differentiation. They also noted that in TINCR deficiency, although epidermal stratification occurs, the expression of key differentiation genes such as *FLG, LOR, ALOXE3, ALOX12B, ABCA12, CASP14* and *ELOVL3* is reduced at both mRNA and protein levels.^18^ Additionally, TINCR lncRNA has been observed to bind to Staufen1 (STAU1), a double-stranded RNA (dsRNA)-binding protein.^18^ STAU1 controls cell differentiation by different mechanisms in different cells. It is implicated in mRNA stabilization in undifferentiated C2C12 myoblasts^29^ and keratinocytes.^18^ Conversely, in adipocytes^30^ and multi-nucleated myotubes,^31^ it can facilitate STAU1-mediated mRNA Decay (SMD) and mRNA degradation. The regulatory effects of TINCR on signaling pathways directly related to cell proliferation, including Wnt/β-catenin,^32^ ERK1/2-SP3,^33^ MAPK,^34^ and mTOR,^35^ demonstrate that this lncRNA can affect different proliferation mechanisms in different tissues. Therefore, in addition to its role in cell differentiation, TINCR may also contribute to the development of psoriasis through its function in cell proliferation.

In our study, the TINCR rs2288947 SNP was significantly associated with an increased risk of psoriasis (e.g., AA vs. GG OR = 1.92), with the AA genotype, the GA+AA model, and the A allele being more frequent in cases than in controls. These findings provide initial evidence that this genetic variant may influence psoriasis susceptibility. Because the observed effects were modest and controls deviated from HWE with a heterozygote-deficit pattern, we interpret these estimates with appropriate caution (QC details in the Supplement). To explore potential regulatory mechanisms underlying this association, we performed a series of bioinformatic analyses using publicly available databases. First, an inquiry into the lncRNASNP database (https://guolab.wchscu.cn/lncRNASNP/) revealed that rs2288947 may alter miRNA-lncRNA interactions by creating novel binding sites for hsa-miR-4443, hsa-miR-4308, hsa-miR-7106-3p, and hsa-miR-6791-5p, while disrupting a known site for hsa-miR-1247-3p. Among these, hsa-miR-4443 is particularly notable, as it has been implicated in regulating cell proliferation and migration through multiple gene targets, and has been associated with various cancerous and non-cancerous diseases.^36^ We note, however, that these are computational predictions and require experimental validation, and thus should be regarded as speculative working hypotheses rather than established mechanisms.

We further examined GTEx-based datasets to assess whether rs2288947 affects TINCR expression (eQTL) or splicing (sQTL). The eQTL analysis revealed that the A allele of rs2288947 is significantly associated with increased TINCR expression in healthy suprapubic skin (*p* = 7.39×10^–9^), following an allele-dose-dependent trend (GG < GA < AA) (Fig. 1). In addition, sQTL analysis demonstrated that rs2288947 significantly alters the alternative splicing pattern of TINCR, suggesting that this variant may influence not only total expression but also the production of distinct transcript isoforms (Fig. 2). Given that lncRNA function is often structure-dependent, and TINCR interacts with RNA-binding proteins such as STAU1, changes in isoform structure may impact its molecular interactions and downstream biological roles. Interestingly, although GTEx-based eQTL analysis indicates that the A allele of rs2288947 is associated with increased TINCR expression in physiologically normal skin, a previous integrative review based on transcriptomic data reported that TINCR is downregulated in psoriatic lesional tissue (fold change = 0.65).^37^ Although this may initially appear contradictory, recent findings suggest that eQTL effects can be highly dependent on the cellular environment, and that their magnitude or direction may differ under inflammatory conditions.^38^ However, to our knowledge, direct evidence showing that TINCR regulation or the effect of rs2288947 specifically changes direction under inflammatory or immunomodulatory conditions is currently lacking. Importantly, GTEx represents bulk, non-lesional skin from healthy donors, and regulatory effects are known to be cell type- and context-dependent. Therefore, GTEx signals should be interpreted as providing baseline regulatory plausibility rather than direct evidence of disease-state mechanisms. Because we lacked genotype-stratified expression data in our cohort (lesional, non-lesional, and control skin), the link between genotype and disease-specific mechanism remains inference-based and will require targeted expression analyses for confirmation.

Taken together, these observations suggest a potentially relevant regulatory context for rs2288947; however, the mechanistic link between genotype and psoriasis susceptibility remains speculative and context dependent. The variant may influence TINCR expression, isoform usage (splicing), and miRNA-mediated post-transcriptional regulation, but these inferences are based on public eQTL/sQTL resources and in silico predictions rather than direct functional or genotype-stratified expression data in our cohort, and should therefore be regarded as hypothesis-generating rather than established effects.

While no significant associations were observed between rs2288947 genotypes and specific psoriasis subtypes (chronic plaque, guttate, erythrodermic, inverse, and pustular), the case-only severity results were not consistent: a significant association appeared with the PASI ≥ 5 threshold, but did not hold under the prespecified continuous severity model. In the absence of treatment covariates and given the therapy sensitivity of PASI, we consider these severity findings exploratory, and the current evidence insufficient to support a robust association between rs2288947 and clinical severity.

Although rs2288947 is not currently included in established Polygenic Risk Score (PRS) models for psoriasis, evaluating its potential contribution to such models may represent a future research direction. This possibility aligns with recent evidence highlighting the contribution of non-HLA genetic variants to clinical phenotype prediction in psoriasis.^39^ However, such evaluation would require replication of the susceptibility association in independent and ethnically diverse cohorts and demonstration that rs2288947 provides additive predictive value beyond established loci.

Another lncRNA known to play a role in keratinocyte proliferation and differentiation is H19. In a study conducted by Li et al., it was reported that H19 lncRNA increased keratinocyte differentiation by weakening the MAPK/ERK signaling pathway via miR-130b-3p and Desmoglein1 (Dsg1).^24^ He et al. suggested that H19 increased keratinocyte proliferation and skin inflammation via the AKT/mTOR pathway by affecting the expression pattern of miR-766-3p.^25^ In our study, however, no statistically significant association was found between the H19 rs217727 SNP and psoriasis susceptibility under genotypic, dominant, or allelic models. This discrepancy with earlier functional studies may be due to population-specific genetic variation, differences in linkage disequilibrium structure, gene-environment interactions, or most notably, limited statistical power. For H19 (rs217727), odds ratios were near unity; however, the 95% Confidence Intervals spanned 1.0 across models and did not exclude modest or model-specific effects, indicating limited precision. Therefore, the absence of statistical significance should be regarded as inconclusive rather than as evidence of no association. Differences in the frequency of the minor allele and genotypic patterns of rs217727 among populations could impact the strength and visibility of any potential genetic associations. Taken together, the absence of a significant association in our cohort does not rule out the functional involvement of H19 in psoriasis pathogenesis. Future studies involving larger and ethnically diverse cohorts, as well as functional investigations such as miRNA binding affinity assays and H19 expression profiling in psoriatic skin, are needed to further elucidate the mechanistic role of this lncRNA and its polymorphisms in disease susceptibility.

This study has several limitations that should be acknowledged. First, although we observed significant associations between rs2288947 and psoriasis susceptibility, we did not investigate the underlying molecular mechanisms through functional assays such as qPCR, luciferase reporter assays, or in vitro expression analyses. We partially addressed this gap by incorporating publicly available GTEx v8 eQTL and sQTL data, which indicate an association between rs2288947 and both TINCR expression and alternative splicing in skin. We caution that these inferences rely on bulk, non-lesional skin from healthy donors; the lack of lesion-specific or single-cell resources limits mechanistic certainty. Second, in controls we observed deviations from Hardy-Weinberg equilibrium (two-sided exact test, *p* < 0.001) for both loci. While high study-wide call rates, absence of case-control differential missingness, and clean cluster separation reduce the likelihood of a pervasive genotyping artifact, subtle population substructure (Wahlund effect) could not be excluded. We did not perform ancestry-informative marker analysis or principal component analysis; therefore, population stratification could not be formally assessed. Accordingly, if present, population stratification could have inflated the observed association between rs2288947 and psoriasis susceptibility, emphasizing the need for replication in independent cohorts. The control-group HWE deviations therefore limit the certainty of the estimates for both loci and further support treating the H19 finding as inconclusive rather than definitively negative. Third, age and sex differed significantly between the case and control groups. The observed age difference between groups was not introduced by study design. Although age, sex, BMI, smoking status, and comorbidities were included in the multivariate logistic regression models, residual confounding by age-related factors, such as cumulative environmental exposures, cannot be completely excluded. Nevertheless, considering the effect size and statistical significance of the association between rs2288947 and psoriasis after adjustment, it is unlikely that the observed association is entirely attributable to the age imbalance. Fourth, due to heterogeneous and limited treatment information, we did not analyze treatment response. Because PASI is therapy-sensitive and standardized treatment covariates were unavailable, residual confounding by therapy cannot be excluded. Within cases, we prioritized a continuous severity model (ln[PASI+1]) with robust standard errors; severity findings were not consistent across specifications and are therefore considered exploratory. Prospective, treatment-adjusted longitudinal studies will be needed to determine whether any genotype-severity link is independent of therapy. In addition, given the exploratory nature of the study, no formal a priori sample size or power calculation was performed. In the absence of such a calculation, the possibility that the available sample had limited power to detect modest genetic effects ‒ particularly for H19 rs217727 ‒ or associations in subgroup analyses (e.g., stratified by sex, clinical subtype, or disease severity) cannot be excluded. Accordingly, some non-significant findings should be regarded as inconclusive rather than definitive null results. Finally, our results are based on a single Turkish cohort, and validation in independent and ethnically diverse populations is needed to ensure generalizability and to assess the potential integration of rs2288947 into polygenic risk prediction models. Future multicenter studies or meta-analyses involving larger and more diverse populations will be essential to confirm these findings and evaluate their broader clinical relevance.

## Conclusion

In conclusion, in this Turkish cohort, TINCR rs2288947 was significantly associated with psoriasis risk, with modest effect sizes. Severity-related findings were inconsistent and exploratory and did not support a robust genotype-severity association. By contrast, H19 rs217727 showed no significant association; given the breadth of the confidence intervals, this result is best viewed as inconclusive. Independent replication and functional studies, particularly genotype-stratified expression and isoform analyses in lesional and non-lesional skin, will be important to establish mechanism, assess generalizability, and determine whether rs2288947 may have future relevance for psoriasis risk prediction.

## Authors’ contributions

Egemen Akgun: The study conception and design; data collection, analysis and interpretation; statistical analysis; writing of the manuscript; critical review of important intellectual content; effective participation in the research guidance; critical review of the literature; final approval of the final version of the manuscript.

Fadime Mutlu Icduygu: The study conception and design; data collection, analysis and interpretation; statistical analysis; critical review of important intellectual content; effective participation in the research guidance; final approval of the final version of the manuscript.

Burak Aksan: The study conception and design; data collection, analysis and interpretation; critical review of important intellectual content; final approval of the final version of the manuscript.

Isil Deniz Oguz: The study conception and design; data collection, analysis and interpretation; critical review of important intellectual content; final approval of the final version of the manuscript.

Sevgi Kulakli: The study conception and design; data collection, analysis and interpretation; critical review of important intellectual content; final approval of the final version of the manuscript.

Ebru Alp: The study conception and design; data collection; analysis and interpretation; statistical analysis; critical review of important intellectual content; effective participation in the research guidance; final approval of the final version of the manuscript.

## Funding

This study was supported by the Scientific Research Projects Committee of Giresun University, Giresun, Turkey (project number: SAĞ-BAP-A-250221-53).

## Conflicts of interest

The authors declare no conflicts of interest.

## Data Availability

The data supporting the findings of this study are available from the corresponding author upon reasonable request.
