# Supplementary material for: A genetic variant of long non-coding RNA TINCR is associated with psoriasis in the Turkish population
Source: Clinics (Sao Paulo). 2026 Sep 30;81:101189. doi: 10.1016/j.clinsp.2026.101189 (PMC13657174; doi:10.1016/j.clinsp.2026.101189)
Supplement: Supplementary file 1 [file mmc1.docx]

**CLINICS-D-25-00153_Supplementary Material**

**Figure S1.** Representative melting-peak (cluster) plots — rs2288947.

Representative melting-peak curves showing allele-specific Tm. Y-axis: −dF/dT (a.u.); X-axis: Temperature (°C).

**Figure S2.** Representative melting-peak (cluster) plots — rs217727.

Representative melting-peak curves showing allele-specific Tm. Y-axis: −dF/dT (a.u.); X-axis: Temperature (°C).

**Table S1. Plate-level call rates and genotype counts — rs2288947**

Per-plate summary of called samples with genotype breakdown (AA/GA/GG).

| **plate** | **attempted_n** | **called_n** | **call_rate** | **AA** | **GA** | **GG** | **flagged_low_qc_n** |
| --- | --- | --- | --- | --- | --- | --- | --- |
| plate1 | 79 | 79 | 1 | 31 | 24 | 24 | 4 |
| plate2 | 56 | 56 | 1 | 12 | 23 | 21 | 3 |
| plate3 | 88 | 88 | 1 | 15 | 30 | 43 | 12 |
| plate4 | 95 | 95 | 1 | 38 | 28 | 29 | 5 |
| plate5 | 87 | 85 | 0.977 | 36 | 23 | 26 | 3 |
| plate6 | 56 | 56 | 1 | 15 | 25 | 16 | 1 |
| plate 7 | 59 | 51 | 0.864 | 18 | 11 | 22 | 8 |
| plate 8 | 34 | 27 | 0.794 | 8 | 14 | 5 | 3 |

**Table S2. Plate-level call rates and genotype counts — rs217727**

Per-plate summary of called samples with genotype breakdown (AA/GA/GG).

| **plate** | **attempted_n** | **called_n** | **call_rate** | **AA** | **GA** | **GG** | **flagged_low_qc_n** |
| --- | --- | --- | --- | --- | --- | --- | --- |
| plate1 | 87 | 87 | 1 | 21 | 24 | 42 | 8 |
| plate2 | 73 | 73 | 1 | 19 | 27 | 27 | 5 |
| plate3 | 12 | 12 | 1 | 3 | 2 | 7 | 1 |
| plate4 | 89 | 89 | 1 | 15 | 31 | 43 | 4 |
| plate5 | 92 | 92 | 1 | 14 | 36 | 42 | 5 |
| plate6 | 25 | 25 | 1 | 1 | 9 | 15 | 0 |
| plate 7 | 85 | 78 | 0.918 | 10 | 40 | 28 | 12 |
| plate 8 | 92 | 81 | 0.890 | 17 | 34 | 30 | 9 |

**Table S3. Case–control differential missingness (called vs missing; Fisher’s test)**

Comparison of called vs missing by case–control group; Fisher p reported in the source sheet if available.

| **SNP** | **Called case** | **Missing case** | **Called control** | **Missing control** | **Fisher p** | **Chi2 p** |
| --- | --- | --- | --- | --- | --- | --- |
| rs2288947 | 284 | 11 | 253 | 5 | 0.309 | 0.210 |
| rs217727 | 284 | 11 | 253 | 7 | 0.633 | 0.492 |

**Table S4a. Flagged borderline calls — rs2288947**

Calls below pre-specified melt-curve thresholds (Resolution < 0.60 and/or Score < 0.90). Sample codes are anonymized.

| **plate** | **Position** | **Sample Name** | **genotype** | **Resolution** | **Score** |
| --- | --- | --- | --- | --- | --- |
| plate1 | A8 | K10 | GG | 0.550 | 0.990 |
| plate1 | B6 | K20 | GA | 0.510 | 0.880 |
| plate1 | B9 | K23 | AA | 0.380 | 0.840 |
| plate1 | C9 | K35 | AA | 0.360 | 1 |
| plate2 | B6 | K115 | GG | 0.510 | 1 |
| plate2 | B9 | K118 | GG | 0.380 | 1 |
| plate2 | H8 | K166 | GG | 0.510 | 1 |
| plate3 | A10 | K178 | GG | 0.890 | 0.890 |
| plate3 | B11 | K191 | GG | 0.590 | 0.970 |
| plate3 | B2 | K182 | AA | 0.590 | 0.990 |
| plate3 | C5 | K197 | GG | 0.500 | 0.970 |
| plate3 | D5 | K209 | GA | 0.590 | 0.880 |
| plate3 | D8 | K212 | AA | 0.820 | 0.890 |
| plate3 | E1 | K217 | AA | 0.740 | 0.890 |
| plate3 | F10 | K238 | GG | 0.570 | 0.980 |
| plate3 | F3 | K231 | GG | 0.840 | 0.880 |
| plate3 | F5 | K233 | GA | 0.740 | 0.870 |
| plate3 | G2 | K242 | GA | 0.610 | 0.890 |
| plate3 | H4 | K288 | GG | 0.610 | 0.890 |
| plate4 | D6 | H51 | GA | 0.590 | 0.880 |
| plate4 | D9 | H54 | AA | 0.380 | 0.840 |
| plate4 | E9 | H68 | AA | 0.750 | 0.720 |
| plate4 | F8 | H80 | AA | 0.740 | 0.820 |
| plate4 | G12 | H105 | GA | 0.580 | 1 |
| plate5 | A11 | H135 | GG | 0.690 | 0.820 |
| plate5 | E3 | H180 | GA | 0.550 | 0.880 |
| plate5 | F8 | H197 | AA | 0.650 | 0.720 |
| plate6 | D12 | H323 | GG | 0.580 | 0.930 |
| plate 7 | A10 | K260 | AA | 0.510 | 1 |
| plate 7 | B2 | K264 | AA | 0.300 | 0.660 |
| plate 7 | C9 | K283 | GA | 0.380 | 1 |
| plate 7 | D10 | K304 | GG | 0.770 | 0.820 |
| plate 7 | D8 | K300 | AA | 0.610 | 0.890 |
| plate 7 | D9 | K301 | GG | 0.560 | 0.990 |
| plate 7 | F9 | H234 | GA | 0.590 | 1 |
| plate 7 | G3 | H240 | GA | 0.610 | 0.890 |
| plate 8 | A11 | H250 | GA | 0.740 | 0.820 |
| plate 8 | E2 | H256 | AA | 0.390 | 0.720 |
| plate 8 | E6 | H260 | GA | 0.650 | 0.720 |

**Table S4b. Flagged borderline calls — rs217727**

Calls below pre-specified melt-curve thresholds (Resolution < 0.60 and/or Score < 0.90). Sample codes are anonymized.

| **plate** | **Position** | **Sample Name** | **genotype** | **Resolution** | **Score** |
| --- | --- | --- | --- | --- | --- |
| plate1 | A8 | K11 | GG | 0.550 | 0.990 |
| plate1 | B6 | K23 | GG | 0.510 | 0.880 |
| plate1 | B9 | K26 | GG | 0.380 | 0.840 |
| plate1 | C9 | K41 | GG | 0.400 | 1 |
| plate1 | D10 | K61 | GG | 0.740 | 0.820 |
| plate1 | F9 | K86 | AA | 0.740 | 0.830 |
| plate1 | G1 | K90 | AA | 0.730 | 0.890 |
| plate1 | G7 | K96 | GA | 0.560 | 0.970 |
| plate2 | B6 | K137 | AA | 0.510 | 1 |
| plate2 | B9 | K141 | GG | 0.380 | 1 |
| plate2 | D10 | K171 | AA | 0.770 | 0.820 |
| plate2 | D9 | K170 | GG | 0.560 | 0.990 |
| plate2 | F9 | K194 | AA | 0.590 | 1 |
| plate3 | C9 | K260 | GA | 0.560 | 0.720 |
| plate4 | E3 | H74 | GG | 0.590 | 0.880 |
| plate4 | E6 | H77 | GG | 0.380 | 0.840 |
| plate4 | F8 | H92 | AA | 0.750 | 0.720 |
| plate4 | G8 | H107 | AA | 0.740 | 0.820 |
| plate5 | A11 | H134 | GA | 0.740 | 0.820 |
| plate5 | E3 | H205 | GG | 0.510 | 0.880 |
| plate5 | E6 | H208 | GG | 0.390 | 0.840 |
| plate5 | F8 | H233 | GG | 0.650 | 0.720 |
| plate5 | G8 | H255 | GG | 0.880 | 0.820 |
| plate 7 | B3 | K199 | GA | 0.690 | 0.760 |
| plate 7 | B5 | K201 | GG | 0.590 | 0.990 |
| plate 7 | C11 | K230 | GA | 0.500 | 0.970 |
| plate 7 | C6 | K225 | GG | 0.300 | 0.720 |
| plate 7 | C9 | K228 | GA | 0.360 | 1 |
| plate 7 | D10 | K241 | GG | 0.500 | 0.970 |
| plate 7 | D5 | K236 | GA | 0.600 | 0.890 |
| plate 7 | D9 | K240 | GG | 0.500 | 0.970 |
| plate 7 | F2 | K301 | GG | 0.600 | 0.890 |
| plate 7 | F4 | K13 | GA | 0.700 | 0.890 |
| plate 7 | G3 | K281 | GG | 0.600 | 0.890 |
| plate 7 | G8 | K286 | GA | 0.600 | 0.890 |
| plate 8 | A4 | H26 | AA | 0.740 | 0.870 |
| plate 8 | C3 | H190 | GA | 0.590 | 0.880 |
| plate 8 | D11 | H244 | GG | 0.740 | 0.870 |
| plate 8 | D3 | H218 | GG | 0.800 | 0.890 |
| plate 8 | D9 | H242 | GG | 0.380 | 0.840 |
| plate 8 | E11 | H283 | GG | 0.750 | 0.720 |
| plate 8 | F2 | H286 | GG | 0.740 | 0.870 |
| plate 8 | F3 | H294 | AA | 0.740 | 0.820 |
| plate 8 | G12 | H332 | GA | 0.580 | 1 |

**Table S5. Hardy–Weinberg metrics in controls**

Control-group genotype counts (AA/GA/GG), allele frequency pA, observed/expected heterozygosity (Hobs/Hexp), inbreeding coefficient (F), and two-sided Hardy–Weinberg p-values from the exact test (HWE_exact_p) and the exact mid-P test (HWE_midP). For consistency, the HWE p-values reported in the main text refer to the exact test.

| **SNP** | **n** | **AA** | **GA** | **GG** | **pA** | **Hobs** | **Hexp** | **F** | **HWE_exact_p** | **HWE_midP** |
| --- | --- | --- | --- | --- | --- | --- | --- | --- | --- | --- |
| rs2288947 | 253 | 70 | 82 | 101 | 0.439 | 0.324 | 0.492 | 0.342 | 5.91E-08 | 4.51E-08 |
| rs217727 | 253 | 53 | 93 | 107 | 0.393 | 0.368 | 0.477 | 0.230 | 0.000344 | 0.000282 |
